# Usage of Spaced Repetition Flashcards to Improve Educational Outcomes in Microbiology

**DOI:** 10.7759/cureus.70994

**Published:** 2024-10-07

**Authors:** Femi Ogunjobi, Seth M Alexander, Lorraine Cramer

**Affiliations:** 1 Microbiology and Immunology, University of North Carolina at Chapel Hill School of Medicine, Chapel Hill, USA; 2 Health Sciences, University of North Carolina at Chapel Hill School of Medicine, Chapel Hill, USA; 3 Internal Medicine and Pediatrics, Vanderbilt University Medical Center, Nashville, USA

**Keywords:** flashcards, microbiology education, scaffolding, spaced repetition flashcards, undergraduate sciences education

## Abstract

*Introduction*: Spaced repetition flashcards and other instructor-made resources are beneficial tools for students in content-dense introductory courses, particularly in the sciences. This study seeks to evaluate whether instructor-made, spaced repetition flashcards affect students' performance on the exams and their self-concept as it pertains to a newly introduced discipline (microbiology).

*Methods*: Students enrolled in a bachelor's level introductory microbiology course utilized a spaced repetition flashcard software to scaffold their review of course material productively. Exam scores and student perceptual data from institutionally validated surveys were then compared using parametric T-testing.

*Results*: While overall performance on the exams was unchanged (*p *= 0.2657), there were significant changes in student perception. Most notably, students' confidence in their ability to succeed improved (*p *= 0.0066), along with their belief that the course made them think like a microbiologist (*p = *0.0011). They also felt that this was an effective instructional approach (*p *= 0.0076).

*Conclusion*: These results suggest that students can better engage with and feel confident in understanding the material presented, even if their exam scores did not drastically improve. Further studies should evaluate how resources like the one trialed here can be implemented to improve students' self-concept and learning.

## Introduction

In microbiology, as in any discipline, there is a steep learning curve often exacerbated by the fact that students may lack a background in fundamental tenets underpinning physical science courses [1]. Instructors often encourage using supplemental resources that model good learning practices and help in the development of essential core concepts. The idea that, with such resources, students will be better able to succeed than if left to their own devices is well described theoretically and in the literature [2]. By providing domain-specific resources to help scaffold and organize the material, instructors lighten the extrinsic cognitive load on students, making it easier to engage with the course's content [3].

Spaced repetition flashcards are one of many tools leveraged to bring the level of content organization found in the classroom to the student in their independent studies. The utilization of spaced repetition flashcards by students to facilitate a well-controlled learning process of course material has been extensively researched and is regarded as both efficient and effective for students of various disciplines and levels of education [4]. Graduate and professional students studying life sciences have used spaced repetition flashcards as a method to improve their learning experience and reduce the perceived difficulty of memorizing extensive content, such as pharmacology and other topics [5-9]. In high schools, teachers have applied spaced repetition models in teaching new languages and have found general improvements in vocabulary retention and recollection [10,11]. Spaced repetition algorithms can maximize exposure to poorly retained content. It also can minimize the re-exposure to learned and retained content. Thus, spaced repetition aids students in optimizing their study time to increase exposure to new material and address specific knowledge gaps. This study explored quantitative performance on exams and qualitative perceptions of students before and after introducing instructor-made spaced repetition flashcards.

## Materials and methods

The study and its methods were conducted at the University of North Carolina at Chapel Hill, spanning two semesters (spring and fall) of an undergraduate introductory microbiology class. The methods of this study were reviewed by the Institutional Review Board of the University of North Carolina at Chapel Hill (UNC), which determined it to be exempt from federal human subject research regulations (UNC IRB Number 19-2126).

Course curriculum and study structure

The course curriculum addresses introductory microbiology topics ranging from pathogenic microorganisms and antimicrobials to human immunology. Two non-cumulative midterm exams and a cumulative final exam are given throughout the course. For the control section, various homework assignments were an ongoing aspect of the course curriculum. These included directed textbook readings associated with guided learning questions (GLQ). In class, students participated in poll questions (Poll Everywhere Inc., San Francisco, CA) and were given templated study guides where students detailed the properties of microorganisms studied throughout class (organism cards). Students are also assigned a weekly online blog post to be written based on prompts related to current course material. Finally, students were also allowed to attend regular instructional sessions with near-peer teachers who reviewed lecture-based content.

The described study is a cohort study completed across two semesters, with each semester comprising a cohort and the second cohort being exposed to the experimental intervention. The experimental section of the course - spaced repetition flashcards - was introduced as another instructor-made resource. Anki (https://apps.ankiweb.net) was the chosen software solution to deliver the flashcard resource since it is a free online program with spaced repetition capabilities. Students were encouraged to review the flashcards regularly and were required to submit a heat map record indicating how often they had used the software. Students were given full credit if they reviewed the flashcards four days in a set weekly assignment period, and partial credit was awarded: 75% for three days, 50% for two days, and 25% if they reviewed on just one day. 

Development and distribution of the resource

**Figure 1:**
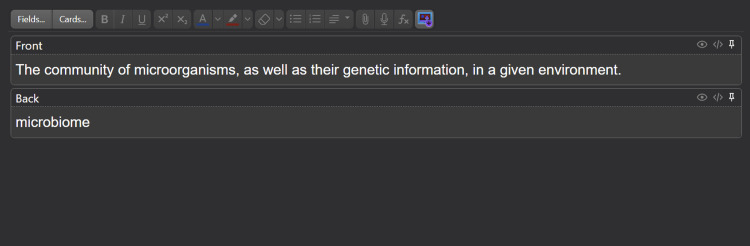
Front-back style Anki flashcard. This image depicts the editing view of an Anki front-back style flashcard. The front of the card, denoted by 'Front,' is the side of the card first exposed to students. The back of the card, denoted by 'Back,' is the side of the card exposed after the front of the card is toggled by a key or mouse click.

**Figure 2:**
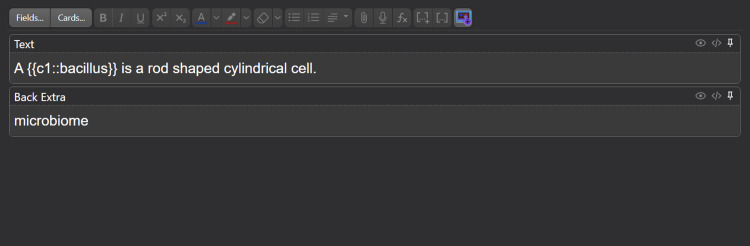
Fill-in-the-blank style Anki flashcard. This image depicts the editing view of an Anki fill-in-the-blank style flashcard. The front of the card, denoted by 'Text,' contains an unrevealed phrase known as cloze text that is revealed after the front of the card is toggled by a key or mouse click.

**Figure 3:**
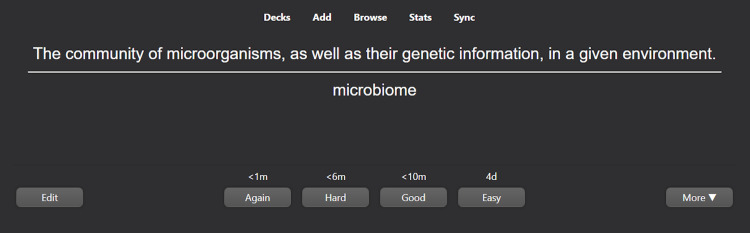
Answer view of an Anki flashcard. This image depicts the answer view of an Anki front-back style flashcard. As with all card styles, students can toggle the card to reappear after a specific timeframe (within one minute, six minutes, 10 minutes, or four days with preset settings applied) based on their perceived ease of recalling its answer. These times increase with each view of the card based on the student's last response to how easy the card was.

In preparing the flashcards, information was primarily sourced from textbook vocabulary terms, images, tables, graphics, and other excerpts from course content. The cards were created by a near-peer instructor and reviewed by the course director and an outside faculty member. The cards were formatted in either a front-back (term-definition) style (Figure 1) or in a fill-in-the-blank style (Figure 2). Ultimately, these cards can be reviewed by students with the answers displayed and reviewed one at a time (Figure 3). The flashcards were partitioned into smaller sub-decks that would be distributed to students at the beginning of each week, allowing students to view and study content in a cumulative or divisional fashion. These resources are available upon request to the corresponding author.

Students' questionnaire: results and analyses

A questionnaire developed by a central faculty group internally at the University of North Carolina and adapted to this course by the study team via Qualtrics XM (Qualtrics, Provost, UT) was distributed to students of both sections shortly before the final exam. The questionnaire collected data on average studying time per week, a breakdown of how that time was spent, perceptions of the course and its resources, perceptions about microbiology, and demographic data. The questionnaire used a Likert scale for qualitative questions. Descriptive and comparative statistical analyses were completed using Stata BE Version 17.0 (StataCorp LLC, College Station, TX).

## Results

The questionnaire was completed by 152 students in the control term and 141 students in the experimental term who consented to their data being analyzed for research purposes. 

Exam performance

The mean performance on the first midterm exam (Exam 1) increased between the control and experimental terms (t = -2.3410, p = 0.0199). The mean performance on all other exams, as well as on a cumulative average of exam scores, did not change significantly between terms. The performance data on the course exams are summarized in Table 1. These findings were consistent when comparing demographic subpopulations (first-generation students, transfer students, and non-white identifying students) between terms. 

**Table 1:** Summary and comparative (based on parametric, unpaired T-test) data on exam performance by the control and experimental populations. * indicates a significant p-value (⍺ = 0.05).

Exam	Population	N	Average Exam Performance (%)	T-value	P-value
Exam 1	Control	152	70.55	-2.3410	0.0199*
	Experimental	141	74.87		
Exam 2	Control	152	74.66	0.7558	0.4504
	Experimental	141	73.29		
Exam 3	Control	149	63.95	-1.5123	0.1316
	Experimental	138	67.31		
Overall Exam Average	Control	152	69.72	-1.1150	0.2657
	Experimental	141	71.67		

Questionnaire results

Students in the two populations each reported, on average, studying for the course between three and four hours a week (t = 0.2911, p = 0.7712). The percentage of this time spent on the instructor-made flashcards increased from a mean of 3.25% among the control population to 29.2% among the experimental population (t =-16.6655, p<0.0001). Notably, there was a significant decrease in the time spent on near-peer teaching, including supplemental instructor (t = 5.4417, p < 0.0001) and peer mentoring sessions (t=6.0335, p<0.0001). A breakdown of study time by percentage across resources, including comparative statistics, can be seen in Table 2. 

**Table 2:** Descriptive and comparative (based on parametric, unpaired T-test) statistics of the percentage of time students reported studying, attending, or using different resources in the control or experimental terms. * indicates a significant p-value (⍺ = 0.05).

Resource	Control Population (%)	Experimental Population (%)	T-value	P-value
Guided Learning Questions	23.86	22.37	0.8069	0.4204
Lecture Recordings	18.05	14.20	1.7408	0.0828
Poll Everywhere Questions	6.13	4.82	1.6949	0.0912
Textbook	19.30	16.63	1.5021	0.1341
Supplemental Instructor Sessions	6.71	2.54	5.4417	<0.0001*
Peer Mentoring Sessions	6.05	1.93	6.03	<0.0001*
Office Hours	0.79	1.69	-2.8408	0.0048*
Student-Made Flashcards	9.11	6.18	2.6239	0.0092*
Instructor-Made Flashcards	3.25	29.21	-16.6655	<0.0001*

Students in the control population (n = 134) reported a mean of 4.20 on a five-point Likert scale regarding the helpfulness of the instructor-made flashcards, referring to the course organism cards that students ultimately completed themselves. Students in the experimental population (n = 139) reported a mean of 4.61 regarding the helpfulness of the instructor-made flashcards (t = -3.8790, p = 0.0001). Between terms, there were significant perceptual shifts regarding the course's instructional structure and influence on the students' thought processes. The perceptual data regarding course resources and organization are summarized in Table 3.

**Table 3:** Comparative data for students' perception of course tools and their utility on a five-point Likert scale for helpfulness or agreeability Scale: 1 = Incredibly Unhelpful/Strongly Disagree up to a score of 5 = Incredibly Helpful/Strongly Agree * indicates a significant p-value (⍺ = 0.05).

Scale	Item or Statement	Control Population	Experimental Population	T-value	P-value
Helpfulness	Lecture	4.09	4.29	-1.5508	0.1220
	Guided Learning Questions	4.23	4.23	-0.0321	0.9745
	Lecture Recordings	4.31	4.18	1.1465	0.2526
	Online Textbook	4.33	4.14	1.7565	0.0801
	Course Blog	2.52	2.97	-4.2573	<0.0001*
	Supplemental Instruction Sessions	4.27	3.88	3.0223	0.0028*
	Peer Mentoring Sessions	3.95	3.78	-1.2373	0.2172
	Student-Made Flashcards	4.03	4.19	-1.3216	0.1875
	Instructor-Made Flashcards	4.20	4.61	-3.8790	0.0001*
Agreeability	"The instructional approach used in the class was effective in helping me learn."	3.43	3.79	-2.6887	0.0076*
	"The class topics, activities, reading, and assignments fit together in a way that helped me learn."	3.91	4.07	-1.5227	0.1289
	"This class made me think like a microbiologist."	3.90	4.24	-3.3029	0.0011*
	"I feel like I learned a lot in this class."	4.29	4.51	-2.3502	0.0194*
	"My work in this class increased my confidence that I can do well in this subject."	3.56	3.92	-2.7383	0.0066*
	"It is important for me to have one-on-one contact with my instructor for this class."	3.32	3.57	-2.1293	0.0341*
	"Some people are just good at microbiology and immunology; you have to be born with the ability."	2.46	2.23	2.1023	0.0364*
	"With enough effort, you can improve your understanding of microbiology and immunology."	3.27	3.43	-2.2204	0.0272*

Correlation between exam performance and flashcard usage

A correlation matrix based on the time spent using the instructor-made flashcards and the average performance on each exam, as well as an overall exam average, was significant only for the first exam (R^2^=0.2241, p<0.0001) and for a positive correlation between exam scores. Subgroup analysis between populations revealed similar results.

## Discussion

While the overall performance on exams between the control and experimental population did not significantly change, the experimental group reported greater comfort and confidence with the content learned throughout the course. These findings were demonstrated by increased student sentiments that becoming good at microbiology and immunology is not innate and, with enough applied effort, an individual can improve their understanding of those disciplines. These improved perceptions can be attributed to the exposure to a highly scaffolded, frequent self-assessment of their understanding, referred to as the testing effect [7,12-15]. The software provided clear and easily accessible benchmarks for comprehension compared to traditional, less structured studying methods such as reading the textbook, attending office hours, or peer tutoring. Comprehension of material in a particular lesson could be easily recognized through the completion or graduation of the corresponding flashcards from a short-term to a long-term learning interval. Therefore, students had a tool to independently gauge their level of preparedness, revise accordingly, and maximize their comfort with the material before exams. Tangentially, the disparity in the perceivable amount of content retained became more pronounced. Scaffolding early in the course likely allows students to proceed farther along in their individual zones of proximal development, coinciding with the improved performance by the experimental population on the first exam [2,3]. However, this advantage wanes after the initial exam, suggesting the benefit may be most useful to orient students to the content before any other structured assessments are delivered. A more directed analysis of this stage of student learning could further describe this phenomenon. 

Though spaced learning has been proven to be a more effective studying strategy than cramming, cramming is still a widely used method that aims to increase a student's exposure to content just shortly before an exam [4]. By encouraging weekly use of flashcards, we strove to reduce last-minute cramming. As a resource that requires long-term usage for retention, spaced repetition resources, such as the flashcard software studied here, yield ineffective results in retention when used to cram content [16-18]. Those who use spaced repetition resources for cramming will have trouble connecting concepts and recollection [15,19]. However, students who cram in preparation for exams can still report the software's potential knowing that if they applied the effort or time necessary in using it based on its designed purpose, it would have yielded positive outcomes in retention. This is an example of how reported positive perceptual changes are not contingent on performing significantly better on assessments.

Implementing the software within the experimental population restructured the time students spent on specific resources throughout the semester. Students spent a lower percentage of their time attending peer tutoring and a higher percentage of their time attending instructor's office hours. The improved confidence exhibited by students may suggest that they are secure and self-assured enough to approach the instructor of the course to discuss the course's content rather than approaching peer tutors who may be a better source for students who are struggling [20-24]. The flashcards helped students identify sections of the content they had the most trouble with and allowed them to precisely communicate those gaps to the instructor, rather than to sources uninvolved in the development of the flashcards. This improved confidence could also explain the increased perception of the course blog if students felt better prepared to synthesize and discuss the course material in a written work. This increased usage during the instructor's office hours and decreased usage in peer tutoring resulted in a similar change in their perceived level of importance and helpfulness, indicating that their utilization by students is directly correlated to their perceived usefulness. 

A primary limitation of the study is the lack of mechanisms to verify the purposeful usage of flashcards or to measure the quality of studying with the software. The distributed flashcard software can be easily clicked through to fulfill the assignment requirements without any legitimate, intentional use for self-assessment or retention. Another limitation of the study is the insufficient number of measured assessments. There is uncertainty on the general performance trend because only two midterm exams were considered in the study. This may be an inadequate number of opportunities to fully adapt and gauge a student's most optimal learning model and resource utilization.

## Conclusions

There was no statistically significant change in the overall exam performance between the control and experimental populations. However, there were significant positive changes in self-concept and utility of other aspects of the course, including student perception of the helpfulness of the instructional approach, their perception of the amount of content they learned, their belief between a link in the comprehension of microbiology and immunology and applied effort, and their deemed importance of one-on-one contact with the instructor. The experimental population had increased confidence and comfort with the course content. Future studies should revise the experiment to measure the quality of resource use against exam performance objectively. Moreover, additional resources should be evaluated to measure how they can improve both students' perceptual changes and learning.
